# The added value of digital breast tomosynthesis in improving diagnostic performance of BI-RADS categorization of mammographically indeterminate breast lesions

**DOI:** 10.1186/s13244-020-0835-2

**Published:** 2020-02-14

**Authors:** Mohammad Abd Alkhalik Basha, Hadeer K. Safwat, Ahmed M. Alaa Eldin, Hitham A. Dawoud, Ali M. Hassanin

**Affiliations:** Department of Radiodiagnosis, Zagazig University, Zagazig, Egypt

**Keywords:** Mammography, Tomography, Breast neoplasm

## Abstract

**Background:**

Mammographic findings are seen more clearly in tomographic images with consequent improvement of Breast Imaging Reporting and Data System (BI-RADS) in categorization of indeterminate breast lesions. This study aimed to evaluate the added value of digital breast tomosynthesis (DBT) to BI-RADS classification in categorization of indeterminate breast lesions after digital mammography (DM) as an initial approach.

**Methods and results:**

We prospectively evaluated 296 women with BI-RADS indeterminate breast lesions (BI-RADS 0, 3, and 4) by DM between January 2018 and October 2019. All patients underwent DBT. Two radiologists evaluated lesions and assigned a BI-RADS category to each lesion according to BI-RADS lexicon 2013 classification using DM, DBT, and combined DM and DBT. The results were compared in terms of main radiological features, diagnostic performance, and BI-RADS classification using histopathology as the reference standard. A total of 355 lesions were detected on DBT and 318 lesions on DM. Thirty-seven lesions were detected by DBT and not seen by DM. The final diagnoses of 355 lesions were 58.3% benign and 41.7% malignant. In comparison to DM, DBT produced 31.5% upgrading and 35.2% downgrading of BI-RADS scoring of breast lesions. DBT reduced number of BI-RADS 3 and 4, compared to DM. All upgraded BI-RADS 4 were malignant. The combination of DBT and DM significantly increased the performance of BI-RADS in the diagnosis of indeterminate breast lesions versus DM or DBT alone (*p* < 0.001).

**Conclusion:**

Adding DBT to BI-RADS improves its diagnostic performance in detection and characterization of mammography indeterminate breast lesions.

## Key points

- DBT produced 31.5% upgrading and 35.2% downgrading of BI-RADS scoring
- The combination of DBT and DM significantly enhanced the BI-RADS performance
- Considering added radiation dose, combined protocol could be limited to suspected lesions

## Background

Breast cancer is the most common cancer among women in the world, accounting for about 12% of all new cancers and 27% of all female cancers [1]. Early detection becomes a critical job to reduce the morbidity and mortality associated with breast cancer [2]. Digital mammography (DM) is the primary breast imaging modality for early detection and diagnosis of breast cancer. However, some limitations persist [3]. One of the substantial limitations of DM is its use in dense breasts [4]. DM has low sensitivity and specificity in women with radiographically dense breast due to decrease contrast between a possible tumor and surrounding breast tissue and summation of tissues may obscure lesions [5]. Digital breast tomosynthesis (DBT) can be expected to overcome this problem by reducing or eliminating the tissue overlap. DBT technology is a modification of a DM unit to allow the acquisition of a three-dimensional (3D) volume of thin section data [4]. The role of DBT for ruling out suspected abnormalities that are identified during screening may be considered an essential diagnostic application [6]. It also allows visualization of cancers not apparent by DM [7]. The more explicit depiction with DBT should allow easier differentiation between benign and malignant lesions [4].

Breast Imaging Reporting and Data System (BI-RADS) was initially developed to allow radiologists to report their level of concern that breast lesions may be missed on DM due to dense tissue but has been widely used in breast cancer research and DM performance research [8, 9].

Several previous studies have shown the high benefits of the addition of DBT in screening programs and the diagnostic setting [10–12]. Mammographic findings are seen more clearly in tomographic images with the consequent improvement of BI-RADS categorization. This fact is reflected, among other things, by the upgrade on BI-RADS classification of malignant lesions not correctly assessed by DM [13], in a better diagnostic performance in dense breasts with BI-RADS 0 findings [14] and in the demonstration of indeterminate lesions (BI-RADS 3 and 4) that are characterized on DM [15]. Consequently, we performed this prospective study to evaluate the added value of DBT to BI-RADS classification in categorization of indeterminate breast lesions (BI-RADS 0, 3, and 4) after DM as an initial approach. Additionally, we made a simple comparison between DBT and DM to test their diagnostic performance in this context.

## Methods

### Study design and population

**Fig. 1:**
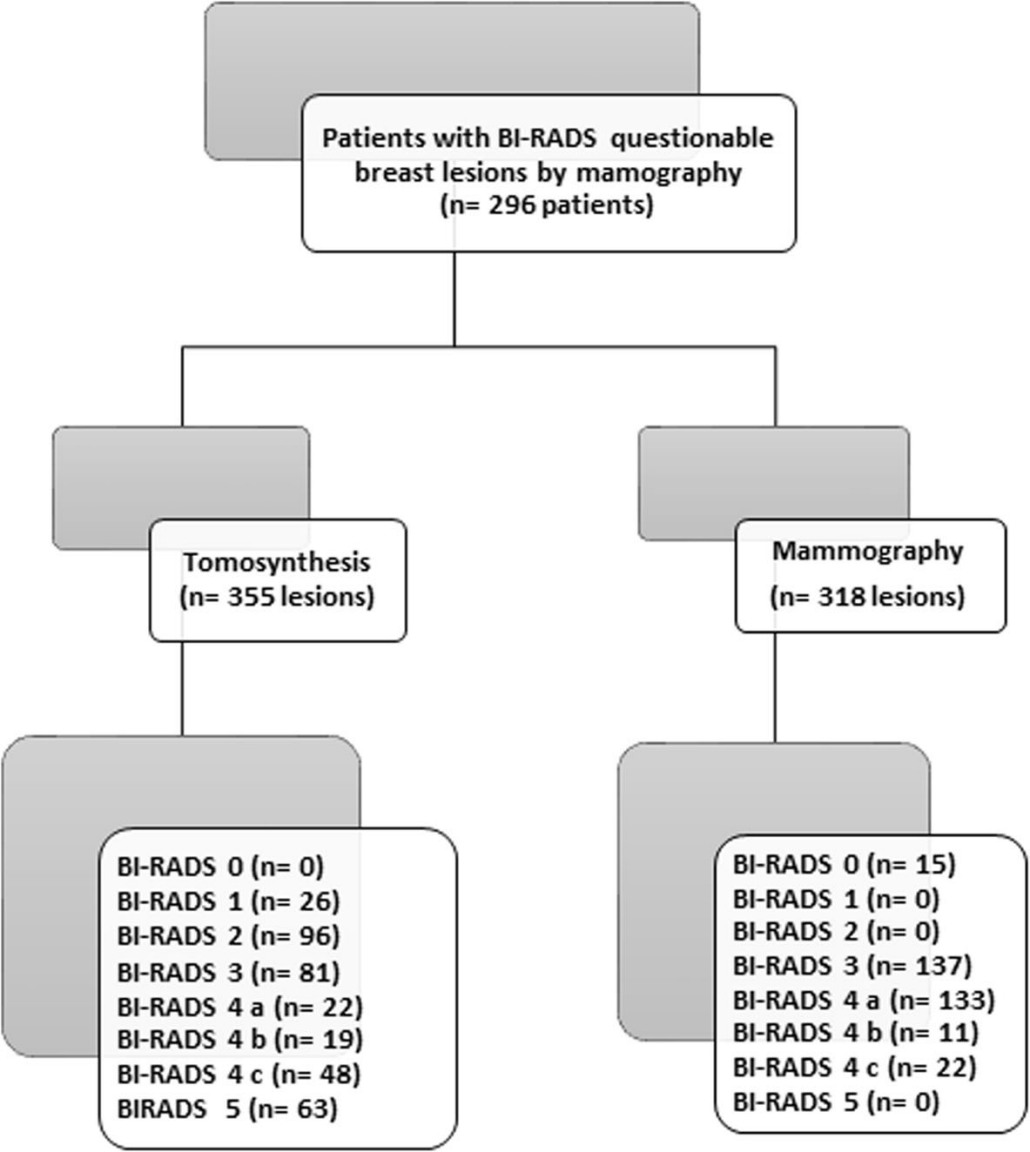
Flow chart of the study population

A prospective study was performed between January 2018 and October 2019. The study was approved by the research and ethical committee, and informed consent was obtained from each patient. Over the 22-month period of the study, three of the authors, who searched in the radiology information system, collected the patients who were categorized as BI-RADS 0, 3, and 4 on DM consecutively to be enrolled in the study and registered the clinical, demographic, and mammographic imaging data of all patients. Inclusion criteria were (i) female ≥ 30 years, (ii) indeterminate breast lesions by DM (BI-RADS 3 and 4), and (iii) dense breast in symptomatic patients (BI-RADS 0). The final cohort of our study included 296 female patients (mean age 46.3 ± 9.4 years, range 32–78 years). The patients’ data are summarized in Table 1. Once enrolled, all patients were requested for second attendance to be subjected to DBT examinations. The flow chart of our study is illustrated in Fig. 1.

**Table 1:** Patients’ data

Variable	Value
Age, years, Mean ± SD (range)	46.3 ± 9.4 (19–78)
Family history	
1st-degree relative	89 (30.1)
2nd-degree relative	59 (19.9)
Negative family history	148 (50)
Site of lesions	
Right breast	170 (57.4)
Left breast	118 (39.9)
Both breasts	8 (2.7)
Clinical presentations	
Asymptomatic	85 (28.6)
Breast lump only	181 (61.2)
Breast lump with breast edema	15 (5.1)
Breast lump with nipple retraction	15 (5.1)
ACR BI-RADS density	
A	15 (5.1)
B	100 (33.8)
C	155 (52.3)
D	26 (8.8)
Final diagnoses of 355 lesions	
Benign	207 (58.3)
Fibrocystic changes	85 (41.1)
Fibroadenomas	63 (30.4)
Normal	26 (12.6)
Postoperative scar	11 (5.3)
Granulomatous mastitis	7 (3.4)
Duct ectasia	7 (3.4)
Abscess	4 (1.9)
Benign phylloides	4 (1.9)
Malignant	148 (41.7)
Invasive ductal carcinoma	104 (70.3)
Invasive lobular carcinoma	26 (17.6)
Ductal carcinoma in situ	11 (7.4)
Mucinous carcinoma	7 (4.7)

Unless otherwise indicated, data are number with the percentage in parenthesis

*SD* standard deviation, *ACR* American college of radiology, *BI*-*RADS* Breast Imaging Reporting and Data System

### The technique of mammography and tomosynthesis

DBT examinations were performed within 2 weeks after DM examinations. All examinations were performed using a full field DM machine with 3D-DBT (Senographe Essential GE healthcare). Each breast was compressed and positioned carefully. Two views for each breast, craniocaudal (CC) and mediolateral oblique (MLO), were taken for both techniques. 3D-DBT involved the acquisition of nine projections with 25° scan angle. The 3D volume of the compressed breast was reconstructed from the 2D projections in the form of a series of images (slices) through the entire breast. Images from both techniques were sent to liquid-crystal display (LCD) screens for reading. No additional views were needed as further processing can be done while viewing the digital images on LCD panels such as zooming, changing contrast, brightness, darkness, inverting the background, and other processing to facilitate lesion detection.

### Image analysis

The previous DM images and the DBT images were transported to the workstation for assessment. The DM and DBT images were separated for interpretation (i.e., the images for DM were interpreted without knowledge of the DBT findings). Two radiologists with 10 years of experience in breast imaging independently reviewed the DM images. After 1 month, the same two radiologists independently reviewed the DBT images. After another month, the same two radiologists independently reviewed DM and DBT images together. Any discrepancies in interpretation were resolved by a third radiologist with over 15 years of experience in breast imaging. The 1-month interval was to diminish the radiologists’ memory bias. All radiologists were blinded to any clinical data or the results of the pathology. The following features obtained at DM and DBT were individually evaluated in each patient: (i) breast density, (ii) site of the lesion, (iii) size of the lesion, (iv) type of lesion (mass or focal asymmetry), (v) mass characters (shape, margin, and density), (vi) asymmetry (simple, focal, global, or developing), (vii) calcifications (morphology and distribution), and (viii) any other suspicious abnormalities.

The four categories of the American College of Radiology (ACR) BI-RADS scale were used to measure mammographic density [16]. The radiologists were requested to assign the BI-RADS category to all detected lesions in each of the two imaging modalities individually according to the BI-RADS lexicon 2013 classification [17]. Finally, each breast lesion had three independent BI-RADS categories (one by DM, one by DBT, and one by combined DM and DBT). In the combined protocol, the radiologists assigned a BI-RADS category based on the combined radiological features from each modality. A feature was considered positive when it was seen in at least one of DM or DBT. The results of DM and DBT for each patient were compared in terms of main radiological features, BI-RADS classification, and diagnostic performance.

### Reference standard

The definitive diagnosis was validated based on histopathologic findings after US-guided biopsy (*n* = 221 patients), stereotactic biopsy (*n* = 32 patients), and surgical mastectomy (*n* = 43 patients). All specimens were examined by two experienced pathologists, and the final results were acquired by consensus. Biopsies were performed to determine the lesion type by the requesting clinician.

### Statistical analysis

Statistical analysis was done using SPSS software version 25 (IBM, 2017). Data were presented in tables and figures. Continuous data were presented as mean and standard deviation. Qualitative data were presented as frequencies and proportions. Pearson’s chi-square (χ^2^) test was used to analyze qualitative data. McNemar test was used to analyze paired qualitative data. The diagnostic performance of DM, DBT, and combined DM and DBT was estimated on a lesion-based analysis. The receiver operating characteristic (ROC) curve analysis was applied to detect the areas under the curve (AUCs). A *p* value of ≤ 0.05 was accepted as statistically significant.

## Results

### Study population

We performed our study on 296 female patients. Every enrolled patient had at least one breast lesion categorized as BI-RADS 0, 3, and 4 on DM. All patients were submitted to DBT. We detected a total of 318 lesions on DM and 355 lesions on DBT. The final diagnoses of 355 lesions were 207 (58.3%) benign and 148 (41.7%) malignant. The most common benign lesion was fibrocystic changes (41.1%), and the most common malignant lesion was invasive ductal carcinoma (70.3%). According to the ACR BI-RADS lexicon for breast density, our patients were divided into four categories: BI-RADS density A, 15 (5.1%) patients; BI-RADS density B, 100 (33.8%); BI-RADS density C, 155 (52.3%); and BI-RADS density D, 26 (8.8 %).

### DM and DBT findings

The DM and DBT findings are presented in Table 2. The DM detected 318 lesions; 148 of them were mass, and 170 were non-mass, while DBT detected 355 lesions; 281 of them were mass, and 74 were non-mass. Of the 26 architectural distortions on DM, 11 revealed underlying masses on DBT. Of the 18 micro-calcifications on DM, 11 revealed underlying masses on DBT. On DBT, five out of 26 cases revealed superposition of normal glandular tissue which were wrongly diagnosed as masses on DM. Thirty-seven lesions were detected by DBT and could not be detected by DM. These lesions were found in the dense breast (BI-RADS density C and D) (*n* = 33) more than non-dense breast (BI-RADS density A and B) (*n* = 4).

**Table 2:** DM and DBT findings

Findings	DM (*n* = 318)	DBT (*n* = 355)
Site of lesion		
Right breast	181 (56.9)	204 (57.5)
Left breast	122 (38.4)	136 (38.3)
Both breasts	15 (4.7)	15 (4.2)
Type of lesion		
Mass	148 (46.5)	281 (79.2)
Asymmetry	111 (34.9)	
Architecture distortion	26 (8.2)	15 (4.2)
Clusters of micro-calcification with no underlying mass	18 (5.7)	7 (2)
Dense breast (BIRADS 0)	15 (4.7)	
Dilated ducts		11 (3.1)
Asymmetrical densities		15 (4.2)
Overlapped glandular tissue	21 (6.6)	26 (7.3)
Characters of mass		
Mass margin		
Obscured on mammography and speculated on tomosynthesis	70 (47.3)	111 (39.5)
Ill-defined	56 (37.8)	44 (15.7)
Well-defined	22 (14.9)	126 (44.8)
Mass shape		
Irregular	67 (45.3)	130 (46.3)
Round	44 (29.7)	92 (32.7)
Oval	19 (12.8)	37 (13.2)
Macrolobulated	18 (12.2)	22 (7.8)
ACR BI-RADS density		
A	15 (4.7)	15 (4.2)
B	107 (33.7)	111 (31.3)
C	170 (53.5)	189 (53.2)
D	26 (8.1)	40 (11.3)

The data are represented as numbers with the corresponding percentages given in parentheses

*DBT* digital breast tomosynthesis, *DM* digital mammography, *BI*-*RADS* Breast Imaging Reporting and Data System, *ACR* American college of radiology

### Assignment of BI-RADS category of breast lesions by DM and DBT

The BI-RADS scoring of breast lesions is summarized in Table 3. The change in individual patient breast lesion owing to DBT, compared to DM, is presented in Table 4. In comparison to DM, DBT produced 31.5% (112/355) upgrading of BI-RADS scoring (4.2% (15/355) in BI-RADS 0, 7.3% (26/355) in BI-RADS 3, 18.8% (56/355) in BI-RADS 4a, 3.1% (11/355) in BI-RADS 4 b, and 1.1% (4/355) in BI-RADS 4 c) and 35.2% (125/355) downgrading of BIRADS scoring (18.9% (67/355) in BI-RADS 3, and 16.3% (58/355) in BI-RADS 4 a). Ninety-three (83%) of upgraded were malignant, and 118 (94%) of downgraded were benign. Sixty of BI-RADS 4 (36.1% (60/166)) were upgraded to BI-RADS 5 by DBT. All upgraded BI-RADS 4 were malignant, and seven of downgraded were malignant. DBT reduced the number of BI-RADS 3 and BI-RADS 4 (81 and 89, respectively), compared to DM (137 and 166, respectively).

**Table 3:** BI-RADS categories of the 355 breast lesions detected on DBT and DM in relation to the final diagnosis

	DBT			DM		
	Malignant	Benign	Total	Malignant	Benign	Total
Not seen	0	0	0	15 (4.2)	22 (6.2)	37 (10.4)
BI-RADS 0	0	0	0	4 (1.1)	11 (3.1)	15 (4.2)
BI-RADS 1	0	26 (7.3)	26 (7.3)	0	0	0
BI-RADS 2	0	96 (27)	96 (27)	0	0	0
BI-RADS 3	16 (4.5)	65 (18.3)	81 (22.8)	30 (8.5)	107 (30.1)	137 (38.6)
BI-RADS 4 a	16 (4.5)	6 (1.8)	22 (6.3)	66 (18.6)	67 (18.9)	133 (37.5)
BI-RADS 4 b	19 (5.4)	0	19 (5.4)	11 (3.1)	0	11 (3.1)
BI-RADS 4 c	34 (9.6)	14 (3.9)	48 (13.5)	22 (6.2)	0	22 (6.2)
BI-RADS 5	63 (17.7)	0	63 (17.7)	0	0	0
Total	148 (41.7)	207 (58.3)	355 (100)	148 (41.7)	207 (58.3)	355 (100)

The data are represented as numbers with the corresponding percentages given in parentheses

*DBT* digital breast tomosynthesis, *DM* digital mammography, *BI*-*RADS* Breast Imaging Reporting and Data System

**Table 4:**
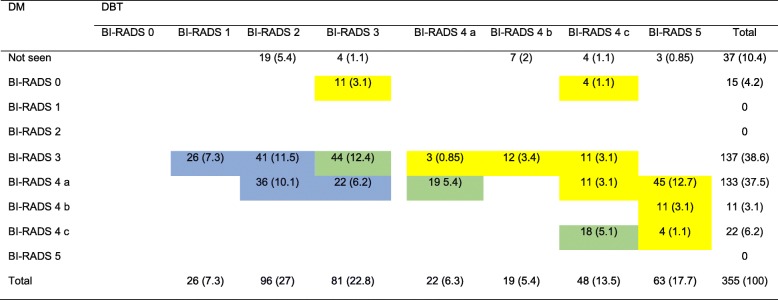
Change in individual breast lesion grading on account of DBT, compared to DM

The data are represented as numbers with the corresponding percentages given in parentheses

The different colors indicate whether DBT upgraded (yellow), downgraded (blue), or kept the grade the same (green) as DM

*DBT* digital breast tomosynthesis, *DM* digital mammography, *BI*-*RADS* Breast Imaging Reporting and Data System

### Diagnostic performance of DM and combined DM + DBT

On a lesion-based analysis, the diagnostic performance of DM, DBT, and combined DBT and DM for breast cancer diagnosis is summarized in Table 5. We considered a combination of BI-RADS 4 and 5 as conclusive for breast cancer diagnosis because the combined BI-RADS 4 and 5 produced higher levels of diagnostic performance than BI-RADS 5 alone. Based on the BI-RADS version 2013, our study showed that BI-RADS with DBT yielded significantly higher accuracy, sensitivity, and specificity than BI-RADS with DM in the diagnosis of breast cancer (*p* < 0.001). The combination of DBT and DM significantly increased the performance of BI-RADS in the diagnosis of breast cancer versus DM or DBT alone (*p* < 0.001). DM had more false-positive and false-negative rates than DBT.

**Table 5:** The diagnostic performance of BI-RADS with mammography, BI-RADS with tomosynthesis, and BI-RADS with combined tomosynthesis and mammography for confident breast cancer diagnosis considered BI-RADS 4 and 5 as predictive of malignancy

Criterion	Mammography		Tomosynthesis		Combined	
	%	95% CI	%	95% CI	%	95% CI
Accuracy	67.3		90		97.5	
Sensitivity	66.9	58.7 to 74.4	89.2	83 to 93.7	98.7	95.2 to 99.8
Specificity	67.6	60.8 to 74%	90.3	85.5 to 94	96.6	93.2 to 98.6
Positive likelihood ratio	2.1	1.7 to 2.6	9.23	6.1 to 14.1	29.2	14.1 to 60.4
Negative likelihood ratio	0.49	0.38 to 0.63	0.12	0.08 to 0.19	0.01	0 to 0.06
Disease prevalence	41.7	36.5 to 47	41.7	36.5 to 47	41.7	36.5 to 47
PPV	59.6	51.8 to 67.2	86.8	80.4 to 91.8	95.4	90.8 to 98.1
NPV	74.1	67.2 to 80.2	92.1	87.5 to 95	99	96.5 to 99.9

*BI*-*RADS* Breast Imaging Reporting and Data System, *AUC* area under curve, *PPV* positive predictive value, *NPV* negative predictive value, *CI* confidence interval

### ROC analyses

**Fig. 2:**
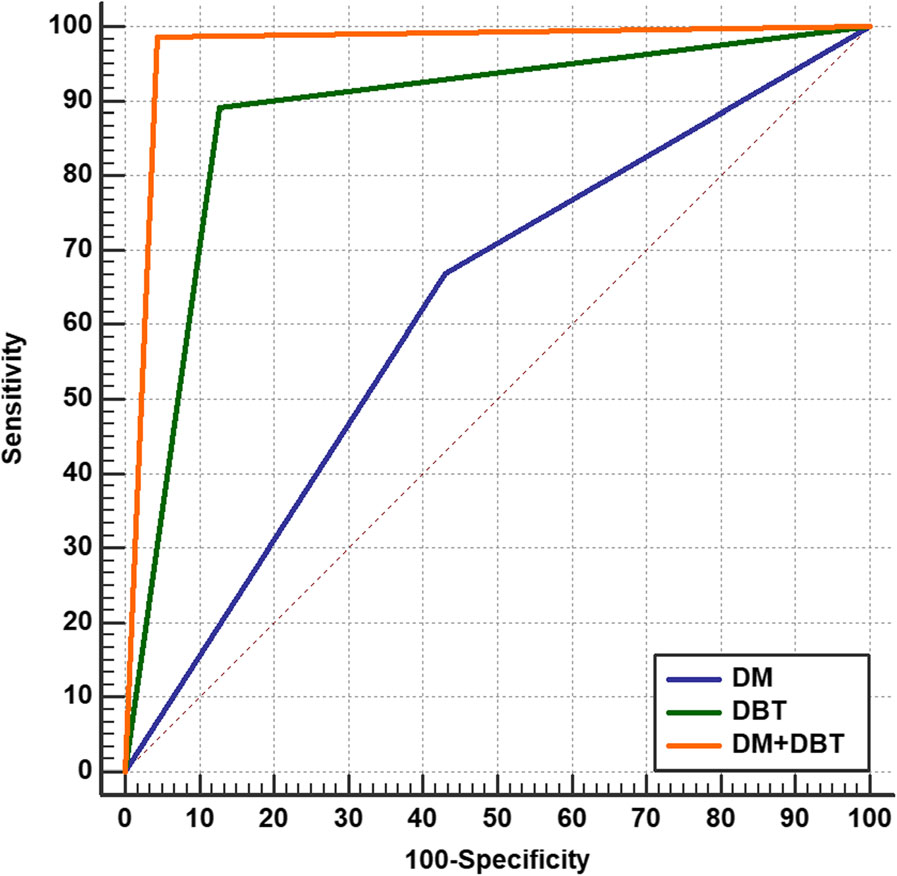
Comparison of the ROC areas of BI-RADS with DM, BI-RADS with DBT, and BI-RADS with combined DM and DBT for breast cancer diagnosis as evidenced by histopathology as the reference standard

We analyzed the data set of the diagnostic performance of BI-RADS with DM, BI-RADS with DBT, and BI-RADS with combined DM and DBT for breast cancer diagnosis using the ROC curve. When the ROC areas were compared, it was found that BI-RADS with DBT was significantly superior to BI-RADS with DM in breast cancer diagnosis (AUC: 0.883 vs. 0.619; *p* < 0.0001; 95% CI 0.214 to 0.313), and the BI-RADS with combined DM and DBT was significantly superior to BI-RADS with DM or BI-RADS with DBT alone (AUC: 0.971; *p* < 0.0001; 95% CI 0.0565 to 0.120) (Fig. 2).

**Fig. 3:**
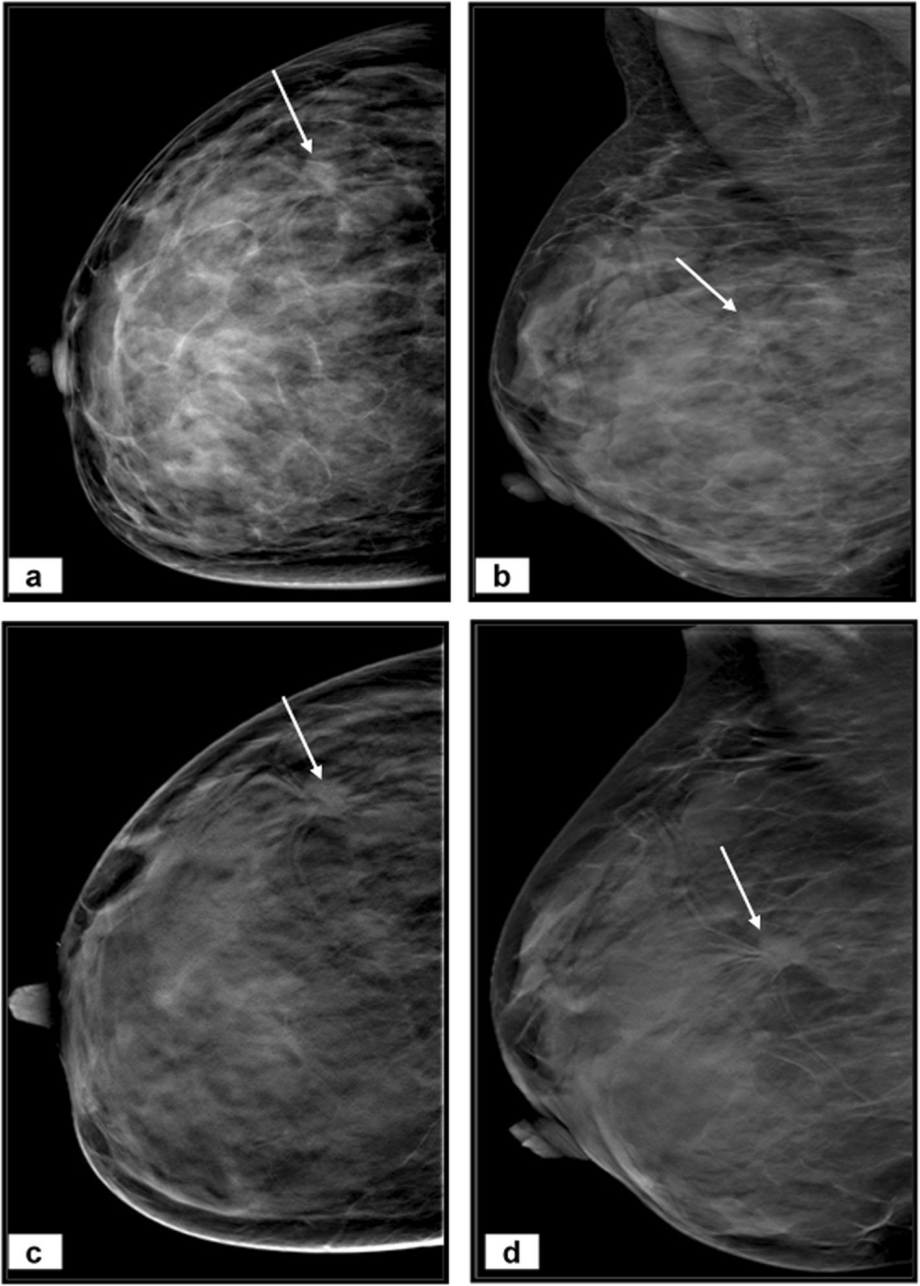
A 35-year-old woman complains of a right breast lump. **a** Craniocaudal and (**b**) mediolateral oblique DM images of right breast reveal extremely dense breast (BI-RADS D) with an outer central area of architectural distortion (arrows). No spiculated masses or microcalcifications. **c** Craniocaudal and (**d**) mediolateral oblique DBT images show a definite lesion with spiculated margins and measures 27 × 25 mm (arrows). The lesion was categorized as BI-RADS 4a by DM and BI-RADS 4c by DBT. Histopathology after surgery revealed invasive lobular carcinoma

**Fig. 4:**
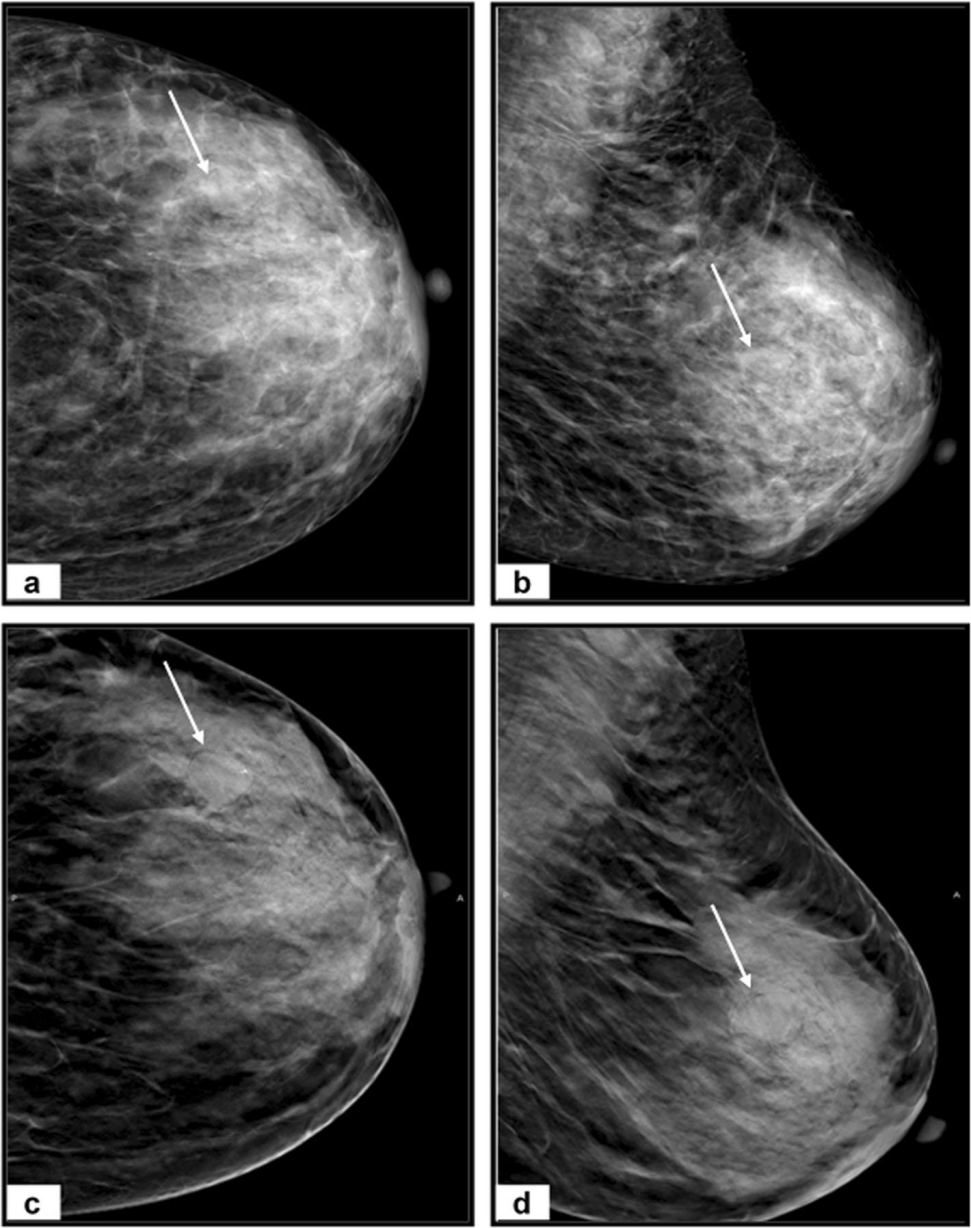
A 48-year-old woman complains of a left breast lump. **a** Craniocaudal and (**b**) mediolateral oblique DM images of left breast reveal heterogeneous dense breast (BI-RADS C) with upper outer quadrant dense lesion with obscured margin (arrows). No spiculated masses or microcalcifications. **c** Craniocaudal and (**d**) mediolateral oblique DBT images show a well-defined rounded medium dense lesion with smooth margins and minute peripheral calcific foci, measures 16 × 16 mm and associated with the characteristic halo sign (arrows). The lesion was categorized as BI-RADS 4c by DM and BI-RADS 2 by DBT. Histopathology after US-guided biopsy revealed simple cyst

**Fig. 5:**
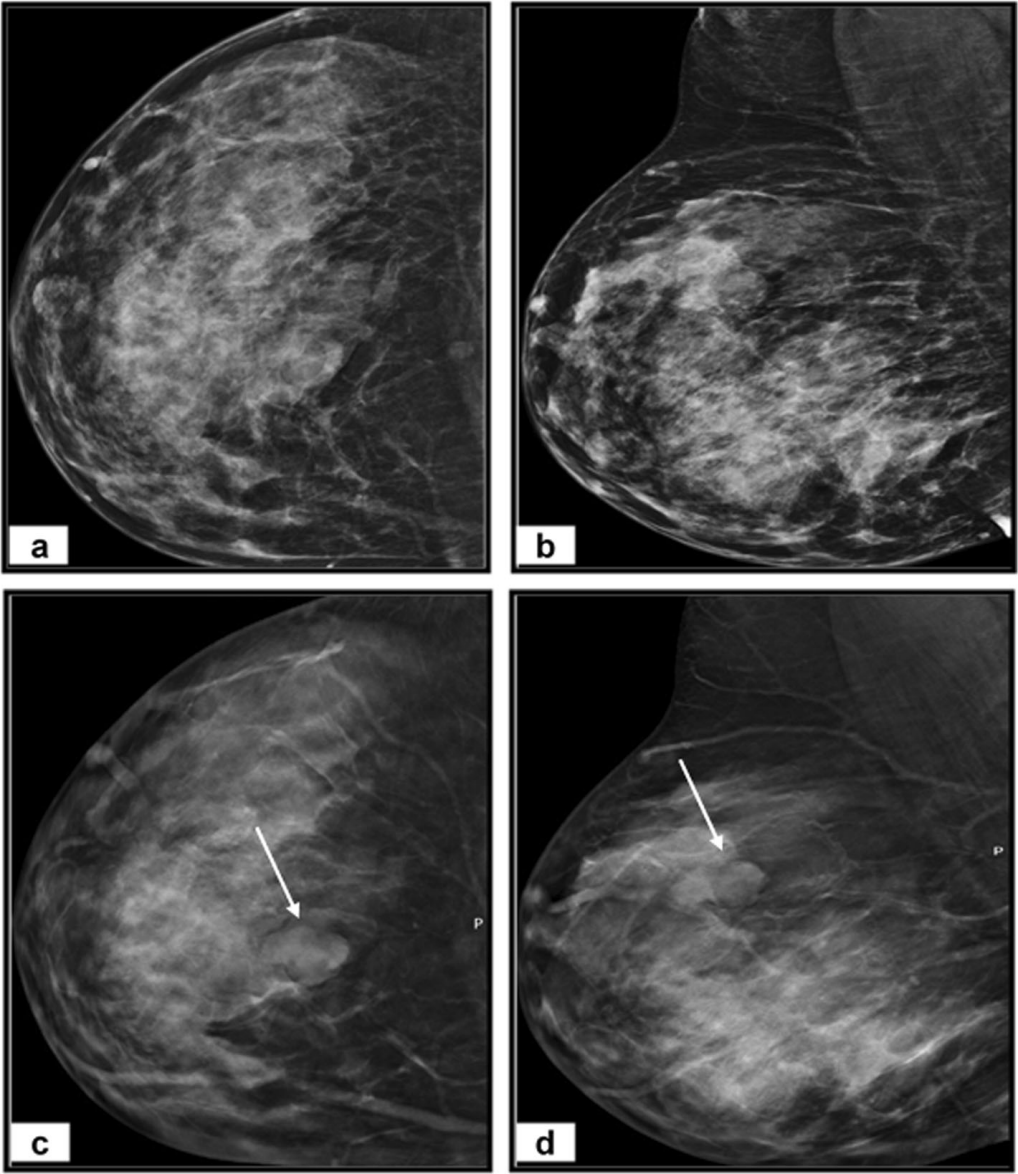
A 36-year-old woman complains of a right breast lump. **a** Craniocaudal and (**b**) mediolateral oblique DM images of left breast reveal heterogeneous dense breast (BI-RADS C). The DM images are inconclusive and need further assessment. **c** Craniocaudal and (**d**) mediolateral oblique DBT images show upper inner quadrant medium density oval-shaped lesion with macrolobulated margins and measures 22 × 20 mm (arrows). The lesion was categorized as BI-RADS 0 by DM and BI-RADS III by DBT. Histopathology after US-guided biopsy revealed fibroadenoma

Our study’s representative cases are illustrated in Figs. 3, 4, and 5.

## Discussion

In this study, we mainly focused on indeterminate BI-RADS categories, because reducing BI-RADS 0, 3, and 4 has pivotal implications for patient care. We aimed to display the added value of DBT to the BI-RADS classification. Few studies evaluated this topic; most of them have investigated category 3 [18, 19], and some have also included category 0 [20]. However, this study focused exclusively on BI-RADS 0, 3, and 4 categories. The overall results of our study confirmed the high diagnostic performance of DBT in the evaluation of indeterminate BI-RADS categories. We found that combined DBT and DM in BI-RADS resulted in a superior sensitivity (98.7%), specificity (96.6%), and accuracy (97.5%) for indeterminate breast lesion categorization than DM or DBT alone; the sensitivity, specificity, and accuracy declined to 66.9%, 67.6%, and 67.3%, respectively, for the DM assessment and 89.2%, 90.3%, and 90%, respectively, for the DBT assessment. Moreover, we found that DBT had a significantly higher sensitivity, specificity, and accuracy than DM in the diagnosis of indeterminate breast lesions. The above findings are matching with that of many previous studies [10, 12, 19, 21–25], which have established that DBT increases the sensitivity and specificity of DM. Consequently, in light of our data, and considering the high diagnostic performance of DBT, we recommend the use of DBT as an additional imaging modality to improve diagnostic accuracy in detecting and characterizing indeterminate breast lesions.

We found that DBT produced a significant change of BI-RADS category in 66.7% lesions with an upgrade in 31.5% lesions (83% were malignant) and a downgrade in 35.2% lesions (94% were benign) in comparison to the DM. This finding agrees with the recent study published by Raghu et al. [12] who have proved that the addition of DBT has been found to change rates of BI-RADS final assessment over time. Similarly, Michell et al. [26] showed a reduction of probably benign cases in 57.8% by an additional DBT.

A remarkable observation of our study was the higher number of lesions identified with DBT than with DM [37 (10.4%)], with BI-RADS 2 lesion representing the greatest number of these missed lesions on DM [19 (51.4%)]. We found that the main cause for missing a lesion on DM was poor visibility due to dense breast parenchyma, tissue overlap, and a radiographically non-conspicuous lesion. In contrast, the DBT decreased interference from overlapping breast tissue and improved lesion conspicuity. These missed lesions on DM cause a significant upgrade of the BI-RADS categories between DM and DBT and subsequently increased diagnostic performance of DBT over DM. This finding indicates that DBT is more accurate than DM in the identification of breast lesion, which is comparable to the previous findings [10, 15, 19]. The increased number of lesions detected on DBT is most probably due to the use of rebuilt images in DBT, as stated by Andersson et al. [13]. These images are obtained from different angles from the breast in a short scanning process and allow the assessment of breast parenchyma where lesions may go unnoticed or less evident due to tissue overlap or increased breast density.

The reduction in the number of BI-RADS 3 and 4 lesions is one of the potential advantages of DBT as some lesions that were categorized as BI-RADS 3 and 4 on DM was upgraded to BI-RADS 5 or downgraded to BI-RADS 1 and 2 based on DBT. This increase in the identification of BI-RADS 3 and 4 lesions by DBT likely result in reduced follow-up of lesions that would not have been identified by DM alone and diminished the requirement for biopsy. These results are comparable to those reported by previous studies [13, 27, 28].

Although DBT has better diagnostic performance than DM, still some breast lesions could not be determined on DBT. On DBT images, improved visualization of a partially or totally smooth boundary in some malignant masses may potentially lead to a misdiagnosis that is false benign diagnosis [29]. Thirty-six of the breast lesions in this study were misdiagnosed on DBT (20 false-positives and 16 false-negatives). Sixteen masses were described as probably benign masses on DBT, but histopathology revealed breast cancer. However, the misdiagnosed lesions on DBT were less than that on DM (67 false-positives and 49 false-negatives). The combined DM and DBT decreased misdiagnosed lesions (seven false-positives and two false-negatives) when compared to DM or DBT alone. Accordingly, our study recommends the use of BI-RADS with a combined DM and DBT protocol, as it improved the BI-RADS performance for diagnosis of indeterminate breast lesions with subsequent potentially better disease management. Similar findings have been seen in various other studies [30–32], in which the addition of DBT decrease in the number of false cases.

When comparing the ROC areas, it was found that DBT is significantly superior to DM in breast cancer diagnosis (AUC = 0.883 vs 0.619; *p* < 0.0001), and the BI-RADS with combined DM and DBT was significantly superior to BI-RADS with DM or BI-RADS with DBT alone (AUC: 0.971; *p* < 0.0001). Similarly, Cai et al. [33] analyzed 79 cases with pathologic results by using ROC curve and showed that the AUC of the combined DM and DBT was greater than that of DM alone (0.914 vs. 0.805). Also, Thomassin et al. [34] calculated AUC by averaging the ROC from four readers; the mean AUC for BI-RADS with combined DM and DBT was higher than that calculated for BI-RADS with DM alone (0.809 vs. 0.685; *p* < 0.01). In contrast, Gennaro et al. [35] concluded that the overall clinical performance with DBT and DM for malignant versus all other cases was not significantly different (AUCs = 0.851 vs. 0.836, *p* = 0.645).

Finally, our study demonstrates that BI-RADS could diagnose the same patient with breast lesions by DBT but not by DM. Thus, we should use DBT in BI-RADS categorization of breast lesions as if we used DM alone as decisive for breast cancer diagnosis; a significant number of breast cancer lesions would be missed by the BI-RADS. The suggested approach for the evaluation of breast cancer lesions according to our results is as follows: first, we perform the DM as a primary imaging modality; if the BI-RAD category provides a confident diagnosis of being benign or malignant (i.e., BI-RADS 1, 2, or 5), the patients go on to the management without further additional imaging. In cases of indeterminate diagnosis (i.e., BI-RADS 0, 3, or 4), we perform DBT, and the BI-RADS category is determined by the combination of DM and DBT findings. We perform the DM initially and not DBT as the former’s lower cost and wide availability.

There are some limitations to our study. First, we focused on indeterminate breast lesions (BI-RADS 0, 3, and 4) and did not consider other BI-RADS categories (BI-RADS 1, 2, and 5). Second, no trial was conducted to analyze the inter-reader agreement in the classification of breast lesions. Third, we did not address the DBT performance in each breast density category. Fourth, the cost-effectiveness and the added radiation dose of combined DM and DBT protocol may be disadvantages of this protocol. Thus, we suggest that the combined protocol be limited to doubted lesions when there remains uncertainty in the BI-RADS category after conducting DM alone.

## Conclusion

Adding DBT to BI-RADS classification improves its diagnostic performance in the detection and characterization of lesions categorized as BI-RADS 0, 3, and 4 by DM.

## Data Availability

The datasets used and/or analyzed during the current study are available from the corresponding author on reasonable request.

## Abbreviations

3D: Three-dimensional
ACR: American College of Radiology
AUC: Area under the curve
BI-RADS: Breast Imaging Reporting and Data System
CI: Confidence interval
DBT: Digital breast tomosynthesis
DM: Digital mammography
LCD: Liquid-crystal display
ROC: Receiver operating characteristic
